# Mosquito Communities Vary across Landscape and Vertical Strata in Indian River County, Florida

**DOI:** 10.3390/pathogens10121575

**Published:** 2021-12-03

**Authors:** Bryan V. Giordano, Anthony Cruz, Daniel W. Pérez-Ramos, Martina M. Ramos, Yasmin Tavares, Eric P. Caragata

**Affiliations:** Florida Medical Entomology Laboratory, Department of Entomology and Nematology, Institute of Food and Agriculture, University of Florida, Vero Beach, FL 32962, USA; anthony.cruz@ufl.edu (A.C.); dperezramos@ufl.edu (D.W.P.-R.); mramos3@ufl.edu (M.M.R.); yasmintavares@ufl.edu (Y.T.); e.caragata@ufl.edu (E.P.C.)

**Keywords:** canopy, stratification, mosquito, vector, GLMM

## Abstract

Mosquito and arbovirus surveillance is essential to the protection of public health. A majority of surveys are undertaken at ground level. However, mosquitoes shelter, breed, and quest for hosts across vertical strata, thus limiting our ability to fully describe mosquito and arboviral communities. To elucidate patterns of mosquito vertical stratification, canopy traps were constructed to sample mosquitoes at heights of 1.5, 5.0, and 8.7 m across three different landscape types in a Florida coastal conservation area. We assessed trapping efforts using individual-based rarefaction and extrapolation. The effects of height, landscape, site location, and sampling date on mosquito community composition were parsed out using permutational ANOVA on a Hellinger-transformed Bray–Curtis dissimilarity abundance matrix. Lastly, a generalized linear mixed effects model (GLMM) was used to explore species-specific vertical patterns. We observed differences in sampling effort and community composition structure across various heights and landscapes. Our GLMM revealed significant effects of trap height for *Aedes taeniorhynchus*, *Anopheles crucians*, *Anopheles quadrimaculatus*, and *Culex coronator,* but not for *Culex nigripalpus*, the ultra-dominant species present in this area. Together these data provide evidence that height and landscape significantly affect mosquito community structures and highlight a need to develop sampling regimes to target specific vector and nuisance species at their preferred height and across different landscape types.

## 1. Introduction

Robust estimates of mosquito population density and arthropod-borne virus transmission rates are essential in informing mitigation efforts and public health messaging campaigns. Rudimentary mosquito collection methodologies rely largely on suction traps baited with light or host-emanations (e.g., carbon dioxide, octenol) since they are affordable, easy to deploy, and capture an appreciable diversity of mosquito species [1]. Nevertheless, adult mosquito trapping is commonly performed at or below shoulder height (≤1.5 m). It has been well documented that questing mosquitoes travel horizontally and vertically through the landscape in search of hosts, oviposition substrates, refugia, or hibernacula. Consequently, the predominance of ground level mosquito surveillance limits our ability to describe mosquito and arboviral communities across vertical strata, where hosts and vectors are known to congregate [2].

Though canopy surveillance has demonstrated utility [3,4,5,6,7], few programs conduct surveillance beyond ground level, and there are limited data available on height bias and mosquito community composition in these overlooked habitats. Furthermore, vertical stratification studies in North America have largely been conducted in the northeastern United States and Canada [3,4,5,6,8,9,10,11,12], with few studies conducted in the southern region of the United States [13,14,15,16,17,18], and even fewer in Florida [19,20].

Florida contains a diverse mosquito community spanning 12 genera and 88 species and has observed a drastic increase in the number of invasive mosquito species introduced over the past decade [21,22,23,24,25]. A dearth of species-specific information pertaining to infection rates and across vertical strata hinders support in the capacity for public mitigation measures in this region. In the current work, we aim to describe mosquito community composition, trap bias, and infection rates by sampling different heights in canopy and understory layers in a Florida conservation area. Sampling habitats that play an important role in mosquito production and persistence have the potential to provide refined richness and abundance estimates and a more efficient WNV surveillance tool. This remains critical, as WNV has been endemic to Florida for more than 20 years, resulting in over 400 human cases. This work will aid public health and mosquito control officials in deciding whether arboreal surveillance and control programs can augment existing methodologies.

## 2. Results

### 2.1. Mosquito Collections

**Figure 1:**
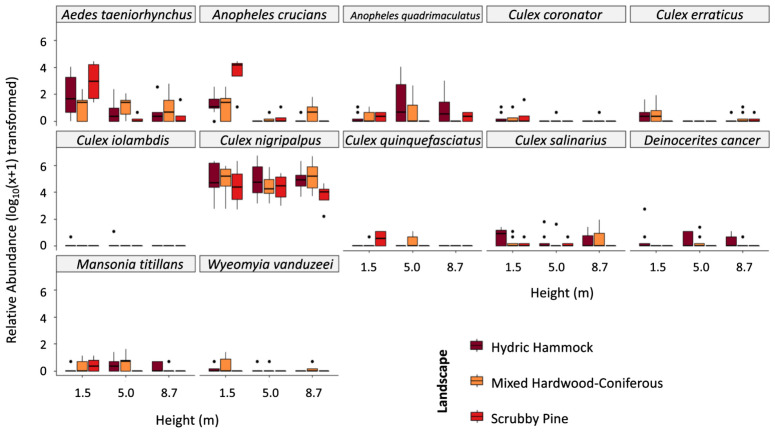
Box plot showing effect of height and landscape on relative abundance of taxa collected in ORCA, Florida from February to April 2021. Dots above box plots represent outliers.

We collected a total of 12,736 adult female mosquitoes from 6 genera including *Aedes albopictus* (representing < 0.1% of total samples collected), *Aedes atlanticus* (<0.1%), *Aedes infirmatus* (<0.1%), *Aedes pertinax* (<0.1%), *Aedes taeniorhynchus* (3.2%), *Anopheles crucians* (2.3%), *Culex atratus* (1.2%), *Culex cedecei* (<0.1%), *Culex coronator* (0.1%), *Culex declarator* (<0.1%), *Culex erraticus* (0.2%), *Culex interrogator* (<0.1%), *Culex iolambdis* (<0.1%), *Culex nigripalpus* (92.0%), *Culex quinquefasciatus* (0.1%), *Culex salinarius* (0.3%), *Deinocerites cancer* (0.2%), *Mansonia titillans* (0.2%), *Wyeomyia mitchelli* (<0.1%), and *Wyeomyia vanduzeei* (0.1%) (Table S1). Relative abundances of taxa across heights and landscapes are presented in Figure 1 and in Table S2 *Culex*, the most abundant taxa across all heights and sites (92.8% of total collections), showed no clear affinity for height. *Culex nigripalpus*, the most abundant taxon across all groups, showed no affinity for height; 1.5 m traps accounted for 35.3% of *C. nigripalpus* collections while the 5.0 m and 8.2 m traps accounted for 34.1% and 30.5%, respectively. Other *Culex* spp. accounted for 0.8% of collections. *Aedes* (3.2% of total collections) and *Anopheles* (3.4% of total collections) were predominantly captured in 1.5 m traps, representing 80% and 65% of genus-level collections, respectively (Table 1).

### 2.2. Trapping Effort across Strata, Landscape, and Site Location

**Figure 2:**
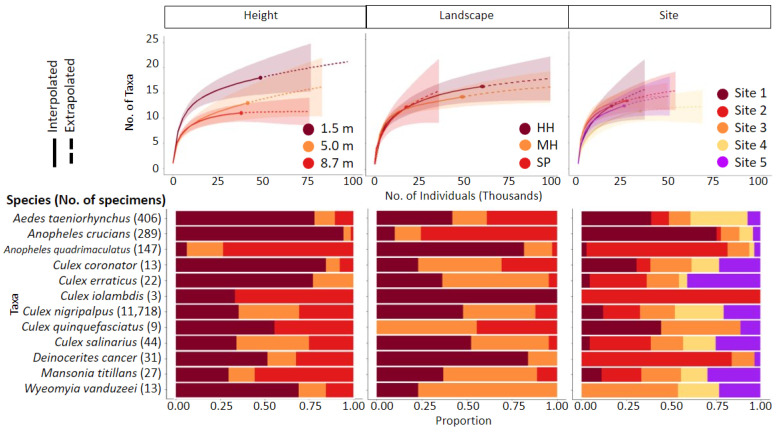
Rarefaction extrapolation curves and bar chart depicting proportional abundances organized by height, landscape, and site location. Generally, richness decreased with height and greater proportions of taxa were collected from the lowest height. HH—hydric hammock, MH—mixed hardwood-coniferous, SP—scrubby pine.

Rarefaction/extrapolation curves and proportions of taxa are presented in Figure 2. In general, suction traps placed at 8.7 m exhibited lower estimates of richness compared to 5.0 and 1. 5 m trap placement; traps placed in hydric hammock observed greater richness estimates than scrubby pine and mixed hardwood-coniferous; and sites 1 through 4 exhibited greater richness estimates than site 5 (Figure 2). The 8.7 m and site 5 interpolated and extrapolated curves approach a clear asymptote at the observed richness (Table 2), indicating that no further sampling is required to improve diversity estimation at these heights. The 1.5 m, 5.0 m, hydric hammock, and mixed hardwood-coniferous curves gradually approach an asymptote at the observed richness, indicating that more sampling is required to improve mosquito community diversity estimation. Rarefaction curves generated for scrubby pine and sites 1 through 4 indicate a requirement for greater sampling. The Simpson (common species) and Shannon (dominant species) diversity estimates indicated that *A. taeniorhynchus* were common at 1.5 m, *A. crucians* was the dominant species in scrubby pine, and *C. nigripalpus* was the common and ultra-dominant species across height, landscape, and site (Table 2).

*Aedes taeniorhynchus* and *A. crucians* showed a clear affinity for scrubby pine and were more abundant at 1.5 m (Figure 1 and Figure 2). *Anopheles quadrimaculatus*, *M. titillans*, and *C. iolamdbis* were captured in greater proportions at 8.7 m. *Culex coronator* was evenly distributed between the three landscapes, but greater proportions were collected at 1.5 m. *Culex erraticus* and *W. vanduzeei* were collected in greater proportions at 1.5 m and in mixed hardwood-coniferous, the former was not collected at 8.7 m and the latter was not observed in scrubby pine collections. *Culex nigripalpus* and *C. salinarius* showed no affinity for height and were obtained in the lowest proportions in scrubby pine. *Culex quinquefasciatus* were not observed at 5.0 m or in hydric hammock collections (sites 2 and 4). *Deinocerites cancer* was collected at all heights but only in hydric hammock and mixed hardwood-coniferous.

### 2.3. Permutational MANOVA

A permutational MANOVA revealed significant interactions between height, landscape (*p* < 0.001), and site location (*p* = 0.032), though composition similarity across the landscapes was not significant after post hoc analyses (Table 3). Trapping at the lowest height affected community structure (*p* < 0.001). Significant changes in community composition were observed between 1.5 m and 5.0 m collections (*p* = 0.004) and 1.5 m and 8.7 m (*p* = 0.003), but not 5.0 m and 8.7 m. Compositions between site 2 and site 4 (*p* = 0.027) and site 2 and site 5 (*p* = 0.020) were significantly different. Time had no distinguishable effect on community composition (Table 3).

### 2.4. Generalized Linear Mixed-Effects Model

Our analysis revealed that height plays an important role in trapping success of *A. taeniorhynchus*, *A. crucians*, *A. quadrimaculatus*, and *C. coronator* (Table S3). *Aedes taeniorhynchus* populations were significantly more abundant in 1.5 m collections (mean ± standard deviation = 15.9 ± 25.2) when compared to 5.0 m (2.4 ± 4.3, incident rate ratio [95% CI] = 0.14 [0.09–0.25], *p* < 0.001) and 8.7 m collections (2.1 ± 3.0, 0.13 [0.08–0.23], *p* < 0.001). *Anopheles crucians* were considerably more abundant in 1.5 m collections (13.7 ± 25.4) when compared to 5.0 m (0.6 ± 1.2, 0.08 [0.04–0.13], *p* < 0.001) and 8.7 m collections (0.2 ± 0.5, 0.02 [0.00–0.04], *p* < 0.001). *Anopheles quadrimaculatus* were substantially less abundant in 1.5 m collections (0.5 ± 0.7) when compared to 8.7 m (5.4 ± 13.0, 5.62 [2.58–12.26], *p* = 0.027). No significant difference was observed between 1.5 m and 5.0 m collections (0.2 ± 0.5, 1.81 [0.86–3.78], *p* = 0.459) or 5.0 m and 8.7 m collections (*p* = 0.231). *Culex coronator* were appreciably more abundant in 1.5 m collections (0.6 ± 1.1) when compared to 5.0 m (0.1 ± 0.2, 0.09 [0.03–0.28], *p* = 0.034) and 8.7 m collections (0.1 ± 0.2, 0.09 [0.03–0.28], *p* = 0.034). Our analysis did not show evidence of significant effects of trap height for *C. erraticus, C. iolambdis*, *C. nigripalpus*, *C. quinquefasciatus*, *C. salinarius*, *D. cancer*, *M. titillans*, or *W. vanduzeei* (Table S3).

We tested 6981 mosquitoes grouped into 305 pools for presence of WNV using an RT-PCR assay (Table S4). We did not detect WNV RNA in the mosquito pools we tested (Table S4).

## 3. Discussion

Our results indicate that height is an important driver of trapping success for several vector and nuisance species in Florida including *A. taeniorhynchus*, *A. crucians*, *A. quadrimaculatus*, and *C. coronator*. *Aedes taeniorhynchus* is an aggressive salt marsh mosquito and major pest in Florida [26]. Seventeen *A. taeniorhynchus* pools have tested positive for the presence of WNV RNA in Florida [27]; however, this species is not considered to be an important vector. In Mexico, *A. taeniorhynchus* has been implicated as the primary vector for dog heartworm (*Dirofilaria immitis*) [28]. *Anopheles crucians* is capable of vectoring *Plasmodium* [29], while *A. quadrimaculatus* has historically been implicated in seasonal malaria transmission foci throughout the United States [30]. *Culex coronator*, an invasive species native to central and south America, first discovered in Florida over a decade ago [21], has demonstrated vector competence for WNV [31] and is now considered a common species in the state [32].

Mosquitoes in the genus *Culex* play an important role in the transmission of West Nile, eastern equine encephalitis, and St. Louis encephalitis viruses. In this study, we collected 10 *Culex* species including *C. nigripalpus* and *C. quinquefasciatus* (important vectors for St. Louis encephalitis virus and WNV in Florida [33,34]), *C. salinarius* and *C. coronator* (competent laboratory vectors of WNV [31,35]), and *C. erraticus* (competent laboratory vector of eastern equine encephalitis virus [36]). Though trapping success was low for *C. quinquefasciatus*, only 9 specimens were recovered, *C. nigripalpus* dominated all collections across height, landscape type, and site, and showed no affinity for height or landscape. *Culex salinarius* abundance in Connecticut was greatest in traps near the ground [4]. In this study, height was not a significant driver of *C. salinarius* abundance. We noted greater trapping success for *C. salinarius* in northeastern Florida using a combination of light and carbon dioxide at ground level [37]. *Culex coronator* were significantly more abundant in ground space collections, though only 13 specimens were recovered in total, potentially introducing bias. This is the first report of host-seeking behavior of *C. coronator* across vertical strata in Florida.

Our permutational MANOVA revealed that mosquito composition was also influenced by landscape (*p* < 0.001) and site location (*p* = 0.032). mixed hardwood-coniferous and hydric hammock assemblages observed a greater richness than scrubby pine. This is likely due to lack of preferred habitat for specialist species. For instance, scrubby pine was devoid of *C. iolambdis*, *D. cancer*, and *W. vanduzeei*; these species utilize a unique ecological niche for one or more developmental stages. For example, *C. iolambdis* oviposit in brackish water (i.e., mangrove habitat) [38]; *D. cancer* utilize land crab burrows for oviposition and refugia [39]; and *W. vanduzeei* utilize ornamental and Florida native bromeliad species (e.g., *Tillandsia utriculate*) for oviposition [40]. Scrubby pine is drier compared to forest habitat types, and the landscape is dominated by a sparse heterogeneous network of sand pines with few bromeliad species. Scrubby pine collections were dominated by host-seeking *C. nigripalpus* and *A. crucians*, permanent or semipermanent freshwater generalists. The lack of preferred habitat in this region suggests mosquito production occurred in the nearby forested and wetland areas or manufactured home and RV community.

All mosquito pools screened for presence of WNV RNA were deemed to be negative (Table S4). We anticipated low detection probabilities given the highly focal nature of WNV epidemics in Florida [33,41] and seasonal patterns of WNV transmission [27]. In Florida, WNV transmission occurs year-round with peak transmission season beginning in July and ending in September [27]. Indian River County participates in a state-wide arbovirus surveillance program utilizing sentinel chickens. During the study period, WNV transmission was not observed in Indian River County or surrounding counties by sentinel chickens, humans (passively reported by physicians), or horses (cases reported to Florida Department of Agriculture and Consumer Services) [27].

Of the 305 pools tested, 123 (40%) contained *C. nigripalpus* (Table S4). During a WNV outbreak in northeastern Florida in 2001, *C. nigripalpus* infection rates were low (1.08 and 7.54 per 1000) and only 1 of 80 chickens in the study seroconverted [33]. Nevertheless, *Culex nigripalpus* populations are abundant year-round in Florida and peak abundance coincides periods of heavy rain [34]. In our collections, *C. nigripalpus* was abundant throughout the trapping period, along with several other disease agents, albeit at much lower abundance.

Though we demonstrate a need for improved and targeted sampling in the canopy and across different landscapes, information on vector use of these habitats is lacking. Knowledge of physiological status (e.g., host-seeking, gravid, blood-engorged) and host-utilization provides valuable information regarding transmission potential and estimates of vectorial capacity. Future work should focus on the physiological status and bloodmeal analysis of mosquitoes captured in the canopy. Furthermore, canopy surveillance and strategic mitigation during the wintertime, when mosquito populations are fragmented due to unsuitable environmental conditions, has potential to maximize control efforts. Reducing vector populations during the winter months could hinder or eliminate arbovirus maintenance cycles and suppress the number of adult mosquitoes emerging in the spring and summer months.

## 4. Materials and Methods

### 4.1. Sampling Locations and Canopy Trap Construction

**Figure 3:**
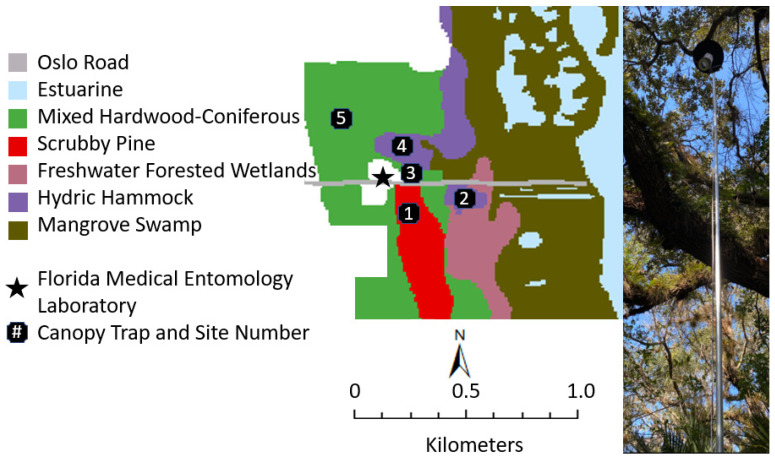
Canopy trap locations in the Oslo riverfront conservation area, Vero Beach, FL. We acquired Cooperative Land Cover v. 3.4 (November 2019, spatial resolution of 10 m) data from the Florida Fish and Wildlife Conservation Commission [42]. Map was produced using ArcGIS 10.3 tool. Photo credit for canopy trap image: B.V.G.

The Oslo riverfront conservation crea (27.586722, −80.375054) located in Vero Beach, Florida contains 298 acres of coastal wetland, hammock, and Florida sand pine scrub habitat navigable by foot along a 1-mile network of trails and intermittent boardwalks. The north side of the conservation area is dominated by cabbage palm (*Sabal palmetto*), southern live oak (*Quercus virginiana*) and other hardwoods. Elevated and drier landscapes to the south are characterized by a preponderance of sand pines (*Pinus clausa*) with a saw palmettos (*Serenoa repens*) understory. Coastal wetlands are guarded by ~350 m of mangrove swamp comprised of red mangrove (*Rhizophora mangle*), white mangrove (*Laguncularia racemosa*), and black mangrove (*Avicennia germinans*). The Florida Fish and Wildlife Conservation Commission recognizes 6 land cover classifications within the conservation area including: estuarine, mixed hardwood-coniferous, scrubby pine, freshwater forested wetland, hydric hammock, and mangrove swamp [42] (Figure 3).

To describe the mosquito community across vertical strata, we sampled mosquitoes from various heights in the canopy and understory layers. We constructed 5 canopy traps in 3 landscapes within the conservation area: site 1: scrubby pine (27.584891, −80.372427), site 2: hydric hammock (27.586525, −80.369171), site 3: mixed hardwood-coniferous (27.587105, −80.372497), site 4: hydric hammock (27.587918, −80.372885), and site 5: mixed hardwood-coniferous (27.588439, −80.374804) (Figure 3). We selected locations with full canopy coverage and flood resilience after heavy rains. Vegetation within freshwater forested wetland and mangrove swamp did not form a complete canopy or reach a height of 8 m, respectively, the latter being inundated with 0.3 to 0.9 m of water.

The base of each trap was a 15.24 cm and 1.5 m diameter Polyvinyl chloride (PVC) tube we inserted 0.9 m into the ground using a post digger. Following this, we combined three 3.33 cm diameter and 3.2 m length fence rails (with tapered ends) to achieve the desired height (~8.7 m). Once leveled, we set the PVC tube and fence rail with fast-setting concrete mix. Mosquito traps were hoisted into the canopy and understory layers using a simple pulley system secured to the top of the fence rail with metal screws (Figure S1).

### 4.2. Mosquito Collection and Identification

Beginning the week of February 8, 2021 and ending April 27, 2021, we trapped mosquitoes at three heights (1.5 m, 5.0 m, and 8.7 m). We utilized a 3 × 5 Latin square design to alternate trapping events between height and 5 sampling locations. Each height was sampled 4 times at each location. Centers for Disease Control and Prevention miniature light traps with the light removed and baited with 2 kg of dry ice (hereby referred to as suction traps; John W Hock Company, Gainesville, FL, USA), were set in the morning between the hours of 8 am and 12 pm and collected 24 h later. Trap contents were transported to the Florida Medical Entomology Laboratory (FMEL, University of Florida|IFAS, Gainesville, FL, USA) on dry ice and placed in a −20 °C freezer for 1 h or until the contents were killed. Mosquitoes were separated from nontargets, speciated using a chill plate and stored at −80 °C until viral testing. Mosquito morphology was assessed under an SMZ745 stereomicroscope (Nikon, Melville, New York, NY, USA) using the keys of Darsie and Morris [43], Darsie and Ward [44], and Burkett-Cadena [45].

### 4.3. RNA Extraction and RT-PCR Assays

RNA extraction and real-time reverse transcriptase polymerase chain reaction (RT-PCR) was performed to determine the presence/absence of WNV RNA at the FMEL BSL-2 facility. Due to a lack of mosquito pool testing data for WNV in Florida we decided to test all species collected. Specimens grouped by date, location, height, and species were placed into 2.0 mL screwcap tubes containing up to 50 individual mosquitoes. To each tube, we added 1 mL of mosquito diluent (88% Dulbecco’s Modified Eagle Medium (Thermo Scientific, Waltham, MA, USA), 10% bovine growth serum (Thermo Scientific, Waltham, MA, USA), 2% Penicillin-Streptomycin (Thermo Scientific, Waltham, MA, USA), and 1 stainless steel bead (Qiagen, Hilden, Germany). Samples were homogenized using a TissueLyser II (Qiagen, Hilden, Germany) following 2 min at 30 Hz. Homogenized samples were centrifuged at 4000 rpm at 4 °C for 20 min. Thereafter, 100 μL supernatant was used for viral RNA extraction using RNeasy Kits (Qiagen, Hilden, Germany). We used a CFX Connect™ real-time PCR detection system (BIO-RAD, Hercules, California, USA) to amplify viral genetic material using the following cycling conditions used by Condotta et al. [46] Each reaction totaled 50 μL including 25.0 μL 2× QuantiTect Probe RT-PCR Master Mix (Qiagen), 13.2 μL RNase-free water, 1.3 μL primer and probe solution (final concentrations: forward/reverse/probe = 1 μM/1 μM/120 nM), 0.5 μL QuantiTect RT Mix (Qiagen), and 10 μL of sample RNA. We used two sets of primers and probes: WN3′NC for initial screening and WNENV for confirmation (Table 1 in Lanciotti et al. [47]). All 96-well PCR plates contained WNV Eg101 (curated by Arbovirus Reference Collection and provided by Reference and Reagent Laboratory of the CDC) as a positive control and ethanol, mosquito diluent, and RNase-free water as negative controls.

### 4.4. Data Analysis

Mosquito count data were organized by date, site, height, and landscape classification in Microsoft Excel 2010 and imported into R programming v. 4.0.2 [48]. We computed richness, Shannon diversity, and Simpson diversity by way of individual-based rarefaction and extrapolation using the “iNEXT” package in R [49,50]. We used a permutational ANOVA following Bray–Curtis distance to determine whether mosquito community composition (site by species abundance matrix) differed between groups organized by height, site location, landscape classification, and sampling date. We selected height, site location, and landscape classification as the explanatory variables and sampling date as a random factor:

Distance matrix ~ land * elevation * site + date

Permutational ANOVA was performed using the *adnois2()* function in the “vegan” package in R [48,51]. Post hoc analyses were performed on all pairwise combinations of variables using the “vegan” *pairwise.adonis()* function and *p*-values were assessed following ‘holm’ correction methodology for multiple tests [51]. The *p*-values were generated following 9999 permutations.

Exploratory data analysis revealed that all species assemblages were zero-inflated, the only exception being *C. nigripalpus* counts. To explore associations of species across vertical strata, we applied a generalized linear mixed effects model (GLMM) using the “glmmTB” package in R [52]. We selected height as the explanatory variable, site location or landscape classification as random factors, and sampling week as a nested random factor. The 1.5 m collections were set as the reference group in all models. We assessed all variable combinations with zero-inflated and non-zero inflated Poisson and negative binomial distributions [53]. Goodness of fit and model selection was based on dispersion parameter (≤1) and lowest Akaike’s Information Criteria and Bayesian Information Criteria. For all species, the model with the lowest AIC and BIC was:

Mosquito count ~ elevation + (1|site/date)

We performed statistical analyses and generated figure graphics in R using the “stats” and “ggplot2” packages, respectively [48,54].

## 5. Conclusions

The canopy represents an overlooked habitat capable of harboring populations of disease agents and provides oviposition substrates, refugia, and protection from aerosolized chemical insecticides. Knowledge of vertical stratification among vector species is of medical and veterinary importance. It is crucial to accurately survey populations and provide control programs with timely and accurate information. Canopy surveillance shows potential in augmenting current approaches and providing improved population density estimates of *Culex* and *Anopheles* in Indian River County. Mosquito community composition in the ORCA was significantly influenced by height, landscape, and site location. Height played a vital role in trapping success for *A. taeniorhynchus*, *A. crucians*, *A. quadrimaculatus*, and *C. coronator*, but not *C. nigripalpus*, the ultra-dominant species in this region.

## Figures and Tables

**Table 1:** Total collected mosquito counts organized by height (m).

Height (m)	Genus					
	*Aedes*	*Anopheles*	*Culex*	*Deinocerites*	*Mansonia*	*Wyeomyia*
1.5	324	4193	282	16	8	10
5.0	47	4027	42	5	4	2
8.2	42	3595	112	10	15	2

**Table 2:** Summary of abundance and alpha-diversity measures (± standard error). HH—hydric hammock, M—mixed hardwood-coniferous, SP—scrubby pine.

Variable	1.5 m	5.0 m	8.7 m	SP	MH	HH	Site 1 (SP)	Site 2 (HH)	Site 3 (MH)	Site 4 (HH)	Site 5 (MH)
Abundance	4833	4127	3776	1803	4913	6020	1803	2643	2428	3377	2485
Richness	18	13	11	12	14	16	12	13	13	11	12
No. Singletons	4	4	1	4	3	3	4	3	2	3	3
Species Density	25.998± 11.660	20.998 ± 11.659	11.250 ± 0.729	19.996 ± 11.656	16.999 ± 4.533	20.499 ± 7.193	19.996 ± 11.656	15.999 ± 4.524	14.000 ± 2.283	12.000 ± 1.870	14.999 ± 4.515
Shannon Diversity	1.835 ± 0.032	1.215 ± 0.017	1.328 ± 0.024	2.103 ± 0.050	1.311 ± 0.018	1.429 ± 0.022	2.103 ± 0.050	1.537 ± 0.034	1.382 ± 0.030	1.298 ± 0.020	1.239 ± 0.023
Simpson Diversity	1.348 ± 0.015	1.064 ± 0.006	1.113 ± 0.009	1.587 ± 0.033	1.095 ± 0.007	1.147 ± 0.007	1.587 ± 0.033	1.193 ± 0.013	1.120 ± 0.011	1.112 ± 0.009	1.070 ± 0.008

**Table 3:** Summary of permutational MANOVA results. Overall significance of all terms *F* (23) = 5.68, *p* < 0.001. ns = not significant after correction.

Variable	R^2^	*F* (df)	*p*-Value	Post Hoc ^1^
Height	0.142	11.89 (2)	<0.001	s:l p_adj_ = 0.003 s:m p_adj_ = 0.004
Landscape	0.038	3.13 (2)	0.007	ns
Site	0.078	6.46 (4)	<0.001	2:5 p_adj_ = 0.020 2:4 p_adj_ = 0.027
Sampling Date	0.416	6.29 (11)	<0.001	ns
Height:Landscape	0.082	3.42 (4)	<0.001	
Height:Site	0.028	2.37 (8)	0.032	
Residual	0.216	(36)		
Total	1.000	(59)		

^1^*p*-values were corrected using ‘holm’ method.

## Data Availability

Data is contained within the article or supplementary material available in the minimum information standard for reporting arthropod abundance data (MIReAD) format (Table S5).

## Supplementary Materials

The following are available online at https://www.mdpi.com/article/10.3390/pathogens10121575/s1, Figure S1: Canopy trap construction, Table S1: Total collected mosquito counts organized by height, landscape, and site, Table S2: Mean number of mosquitoes per trap night and richness by height and landscape, Table S3 Results of generalized linear mixed effect models of mosquito species abundances captured at different heights, Table S4: Summary of mosquito pool testing for presence of WNV by an RT-PCR assay, Table S5: All collection data presented in the minimum information standard for reporting arthropod abundance data [55].
