# Supplementary material for: Mosquito Communities Vary across Landscape and Vertical Strata in Indian River County, Florida
Source: Pathogens. 2021 Dec 3;10(12):1575. doi: 10.3390/pathogens10121575 (PMC8708810; doi:10.3390/pathogens10121575)
Supplement: Supplementary file 1 [file pathogens-10-01575-s001.zip › pathogens-1452457-suppelmentary( Table S1-4, Figure S1).pdf]

pathogens

MDPI

Table S1. Total collected mosquito counts organized by height, landscape, and site. Site numbers correspond to Figure 1. HH – Hydric Hammock, MH – Mixed Hardwood-Coniferous, SP – Scrubby Pine.

	Height (m)			Landscape			Site				
Species	1.5	5.0	8.7	HH	MH	SP	1	2	3	4	5
Aedes albopictus	2	0	0	1	0	1	1	0	0	1	0
Aedes atlanticus	3	0	0	2	0	1	1	0	0	2	0
Aedes infirmatus	1	0	0	0	0	1	1	0	0	0	0
Aedes pertinax	1	0	0	1	0	0	0	1	0	0	0
Aedes taeniorhynchus	317	47	42	170	78	158	158	40	49	130	29
Anopheles crucians	273	12	4	29	42	218	218	7	30	22	12
Anopheles quadrimaculatus	9	30	108	120	23	4	4	116	18	4	5
Culex atratus	0	1	0	1	0	0	0	1	0	0	0
Culex cedecei	3	0	0	3	0	0	0	3	0	0	0
Culex coronator	11	1	1	3	6	4	4	1	3	2	3
Culex declarator	0	1	0	0	1	0	0	0	1	0	0
Culex erraticus	17	5	0	8	13	1	1	7	4	1	9
Culex interrogator	0	1	0	0	1	0	0	0	0	0	1
Culex iolambdis	1	0	2	3	0	0	0	3	0	0	0
Culex nigripalpus	4141	4000	3577	5617	4695	1406	1406	2417	2293	3200	2402
Culex quinquefasciatus	5	0	4	0	5	4	4	0	4	0	1
Culex salinarius	15	18	11	23	19	2	2	15	8	8	11
Deinocerites cancer	16	5	10	26	5	0	0	26	4	0	1
Mansonia titillans	8	4	15	10	14	3	3	6	6	4	8
Wyeomyia mitchelli	1	0	0	0	1	0	0	0	1	0	0
Wyeomyia vanduzeei	9	2	2	3	10	0	0	0	7	3	3
Damaged specimens	16	69	8	47	44	2	2	0	7	3	3
Males	0	1	0	0	1	0	0	0	0	1	0

Table S2. Mean number of mosquitoes per trap night and richness by height and landscape. Standard deviation is in parenthesis. Values followed by the same letter are not significant different ($p < 0.05$). .

	Height (m)			Landscape		
Species	1.5	5.0	8.7	Hydric Hammock	Mixed Hardwood-Coniferous	Scrubby Pine
Aedes albopictus	0.0 (0.0)	0.0 (0.0)	0.1 (0.3)	0.0 (0.2)	0.0 (0.0)	0.1 (0.3)
Aedes atlanticus	0.2 (0.5)	0.0 (0.0)	0.0 (0.0)	0.1 (0.4)	0.0 (0.0)	0.1 (0.3)
Aedes infirmatus	0.1 (0.2)	0.0 (0.0)	0.0 (0.0)	0.0 (0.0)	0.0 (0.0)	0.1 (0.3)
Aedes pertinax	0.1 (0.2)	0.0 (0.0)	0.0 (0.0)	0.1 (0.2)	0.0 (0.0)	0.0 (0.0)
Aedes taeniorhynchus[1]	15.9 (25.2)	2.4 (4.3)	2.1 (3.0)	7.1 (15.0)	3.3 (3.9)	13.2 (28.1)
Anopheles crucians[1]	13.7 (25.4)	0.6 (1.2)	0.2 (0.5)	1.2 (2.7)	1.8 (2.9)	18.2 (32.5)
Anopheles quadrimaculatus[1]	0.5 (0.7)	1.5 (4.3)	5.4 (13.0)	5.0 (12.2)	1.0 (2.7)	0.3 (0.5)
Culex atratus	0.0 (0.0)	0.1 (0.2)	0.0 (0.0)	0.1 (0.2)	0.0 (0.0)	0.0 (0.0)
Culex cedecei	0.2 (0.7)	0.0 (0.0)	0.0 (0.0)	0.1 (0.6)	0.0 (0.0)	0.0 (0.0)

Culex coronator[1]	0.6 (1.1)	0.1 (0.2)	0.1 (0.2)	0.1 (0.4)	0.3 (0.6)	0.3 (1.2)
Culex declarator	0.0 (0.0)	0.1 (0.2)	0.0 (0.0)	0.0 (0.0)	0.0 (0.2)	0.0 (0.0)
Culex erraticus[1]	0.9 (1.6)	0.3 (.6)	0.0 (0.0)	0.3 (0.9)	0.5 (1.3)	0.1 (0.3)
Culex interrogator	0.0 (0.0)	0.1 (0.2)	0.0 (0.0)	0.0 (0.0)	0.0 (0.2)	0.0 (0.0)
Culex iolambdis[1]	0.1 (0.2)	0.0 (0.0)	0.1 (0.4)	0.1 (0.4)	0.0 (0.0)	0.0 (0.0)
Culex nigripalpus[1]	207.0 (185.9)	200.0 (211.5)	178.8 (222.2)	234.0 (236.6)	195.6 (187.9)	117.2 (148.9)
Culex quinquefasciatus[1]	0.3 (0.6)	0.0 (0.0)	0.2 (0.5)	0.0 (0.0)	0.2 (0.5)	0.3 (0.8)
Culex salinarius[1]	0.8 (1.1)	0.9 (1.8)	0.6 (1.4)	1.0 (1.4)	0.8 (1.7)	0.2 (0.4)
Deinocerites cancer[1]	0.8 (3.4)	0.3 (0.6)	0.5 (0.9)	1.1 (3.1)	0.2 (0.7)	0.0 (0.0)
Mansoni titillans[1]	0.4 (0.7)	0.2 (0.4)	0.8 (1.1)	0.4 (0.7)	0.6 (1.0)	0.3 (0.6)
Wyeomyi mitchelli[1]	0.1 (0.2)	0.0 (0.0)	0.0 (0.0)	0.0 (0.0)	0.1 (0.2)	0.0 (0.0)
Wyeomyi vanduzeei[1]	0.5 (0.9)	0.1 (0.3)	0.1 (0.3)	0.1 (0.3)	0.4 (0.9)	0.0 (0.0)
Richness[2]	5.3 (1.7)	3.3 (1.6)	3.4 (1.9)	4.2 (1.9)	4.1 (1.8)	3.2 (2.2)

[1]Significant difference was assessed by generalized linear mixed effects model .

Table S3. Results of generalized linear mixed effect models of mosquito species abundances captured at different heights. ZINB, zero-inflated negative binomial; ZIP, zero-inflated poisson.

	**Conditional Model**			
**Species**	**Variable**	**Coefficient (se)**	**z value**	**Pr(>|z|)**
Ae. taeniorhynchus ZINB	Intercept	2.762 (0.366)	7.547	< 0.001
	1.5 m	Ref.		
	5.0 m	–1.909 (0.535)	–3.568	< 0.001
	8.7 m	–2.021 (0.537)	–3.762	< 0.001
An. crucians ZINB	Intercept	2.074 (0.530)	3.912	< 0.001
	1.5 m	Ref.		
	5.0 m	–2.631 (0.619)	–4.321	< 0.001
	8.7 m	–4.046 (0.713)	–5.679	< 0.001
An. quadrimaculatus ZINB	Intercept	–0.850 (0.730)	–1.165	0.244
	1.5 m	Ref.		
	5.0 m	0.591 (0.798)	0.740	0.459
	8.7 m	1.726 (0.780)	2.212	0.027
Cx. coronator ZIP	Intercept	0.610 (0.376)	1.623	0.105
	1.5 m	Ref.		
	5.0 m	–2.429 (1.143)	–2.126	0.034
	8.7 m	–2.429 (1.143)	–2.126	0.034
Cx. erraticus ZIP	Intercept	–0.954 (0.548)	–1.743	0.081
	1.5 m	Ref.		
	5.0 m	–1.14 (0.709)	–1.605	0.108
	8.7 m	-	-	-
Cx. iolambdis ZIP	Intercept	–6.871 (5.824)	–1.180	0.238
	1.5 m	Ref.		
	5.0 m	-	-	-
	8.7 m	0.965 (1.423)	0.678	0.498
Cx. nigripalpus ZIP	Intercept	4.787 (0.250)	19.133	<0.001
	1.5 m	Ref.	-	-
	5.0 m	–0.011 (0.354)	–0.032	0.975
	8.7 m	–0.186 (0.354)	–0.525	0.599
Cx. quinquefasciatus ZIP	Intercept	0.024 (0.061)	0.036	0.971
	1.5 m	Ref.		
	5.0 m	-	-	-
	8.7 m	–0.327 (0.810)	–0.403	0.687

Cx. salinarius ZIP	Intercept	0.393 (0.369)	1.066	0.286
	1.5 m	Ref.		
	5.0 m	0.494 (0.461)	1.072	0.284
	8.7 m	0.229 (0.584)	0.392	0.695
De. cancer ZIP	Intercept	−3.642 (1.510)	−2.412	0.016
	1.5 m	Ref.		
	5.0 m	0.122 (1.094)	0.112	0.911
	8.7 m	0.937 (1.024)	0.916	0.360
Ma. titillans ZINB	Intercept	−0.916 (0.392)	−2.340	0.019
	1.5 m	Ref.		
	5.0 m	−0.693 (0.657)	−1.055	0.291
	8.7 m	0.629 (0.498)	1.261	0.207
Wy. vanduzeei ZINB	Intercept	−1.147 (0.718)	−1.598	0.110
	1.5 m	Ref.		
	5.0 m	−1.601 (0.898)	−1.782	0.075
	8.7 m	−1.549 (0.891)	−1.739	0.082

Table S4. Summary of mosquito pool testing for presence of WNV by an RT-PCR assay. .

Species	Total Number of mosquitoes tested	Number of Pools	Average Pool Size	No. Positive Pools
Aedes albopictus	2	2	1.0	0
Aedes atlanticus	3	2	1.5	0
Aedes infirmatus	1	1	1.0	0
Aedes pertinax	1	1	1.0	0
Aedes taeniorhynchus	412	39	10.6	0
Anopheles crucians	289	26	11.1	0
Anopheles quadrimaculatus	147	21	7.0	0
Culex atratus	1	1	1.0	0
Culex cedecei	6	2	3.0	0
Culex coronator	13	7	1.9	0
Culex declarator	1	1	1.0	0
Culex erraticus	22	12	1.8	0
Culex interrogator	1	1	1.0	0
Culex iolambdis	3	2	1.5	0
Culex nigripalpus	5953	123	48.4	0
Culex quinquefasciatus	9	6	1.5	0
Culex salinarius	45	18	2.5	0
Deinocerites cancer	31	11	2.8	0
Mansonia titillans	27	19	1.4	0
Wyeomyia mitchelli	1	1	1.0	0
Wyeomyia vanduzeei	13	9	1.4	0

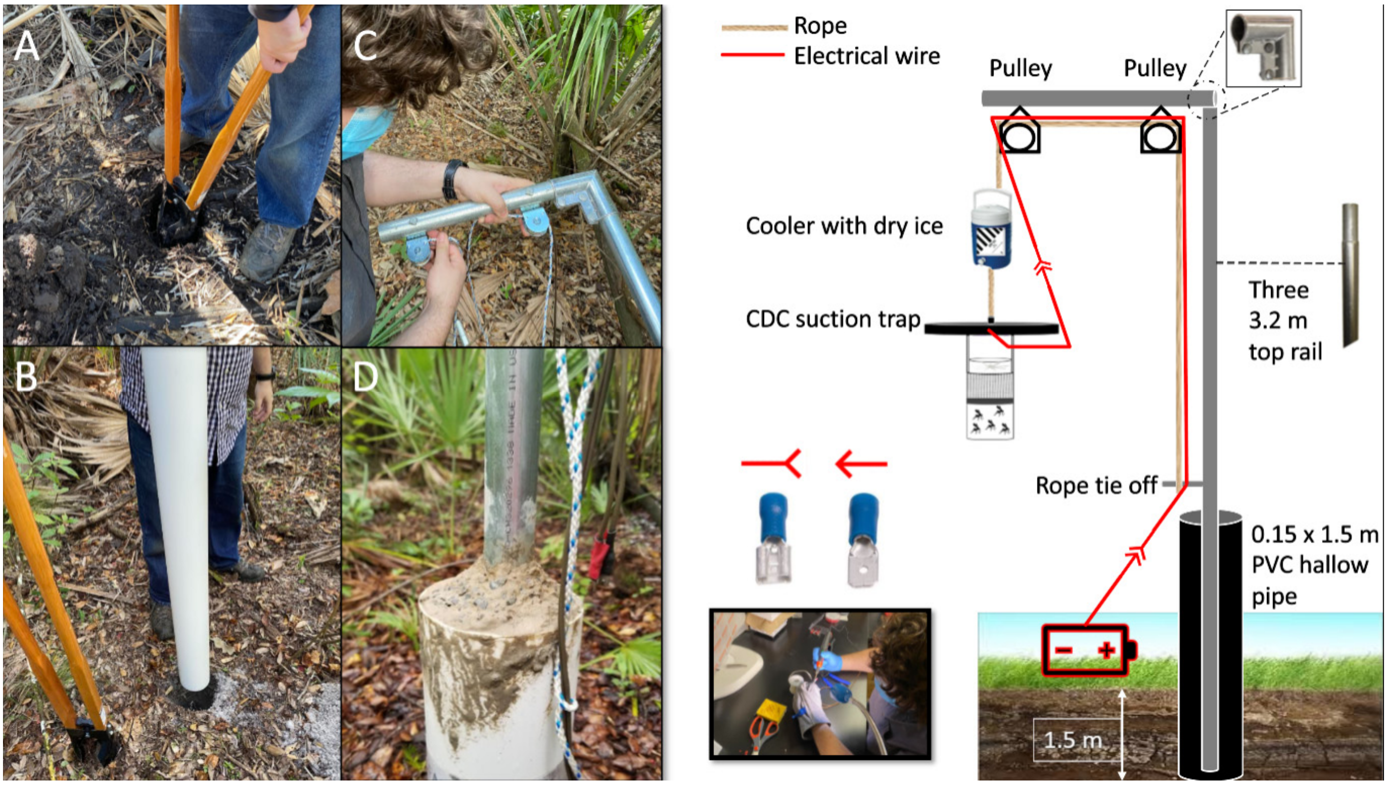

Figure S1. Canopy trap construction.(**A**) A 0.9 m hole was dug using a fence-post digger. (**B**) A 1.5 m PVC pipe was secured in place using sand and a dowel was used to pack the sand. (**C**) We assembled 3–3.2 m top rails and secured 0.3 m of metal pipe to the top using a gate hinge. Two pullies were attached using metal screws and we fed the rope and electrical wire through each pully. (**D**) The rail system was placed inside the PVC pipe and secured in place as previously described until the PVC pipe was filled with 2/3 of packed sand. The remaining top 1/3 was filled with quick dry cement following manufacturer's instructions. .
